# Mercy Hospital Dispensary

**Published:** 1874-10-15

**Authors:** F. H. Davis


### Clinical Reports.

### MERCY HOSPITAL DISPENSARY.—SPECIAL CLINIC FOR DISEASES OF THE THROAT AND CHEST.

IN CHARGE OF F. H. DAVIS, M. D.

NASAL CATARRH.— Numerous cases of this very common and troublesome affection present themselves at this season of the year. They are of all grades of severity, from the recent acute inflammation, characterized by a free copious mucous or slightly purulent discharge to the old chronic *ozæna*, accompanied by the fetid purulent discharge, and chronic thickening of the nasal mucous membrane. The partial or complete occlusion of one or both nostrils by the accumulation of hardened secretion, and the swelling of the parts is of frequent occurrence, also partial deafness from closure of eustachian tubes. In most chronic cases the inflammation spreading down over the fauces involves more or less the larynx and larger bronchial tubes, producing hoarseness and slight cough with expectoration.

As met with in dispensary practice many of these cases present an evident syphilitic element. A broadened, thickened condition of the bridge of the nose from periosteal inflammation is a common evidence of this taint, also the destructive ulcerated patches in the nares, or more frequently on the fauces bear evidence to the same effect.

The treatment that we have been in the habit of pursuing in these cases of nasal catarrh is very simple, but apparently quite as efficient and successful as any that has been devised. The nasal passages are directed to be cleansed once or twice each day, either by the nasal douche or syringe; a solution of salt and water being used for the purpose.

The following solution is directed:

℞ Iodine Cryst., grs. xii.
Chloroform, ------ ℥ i.

Mix.

To be inhaled two or three full breaths at a time, through either nostril, several times through the day. Slight or recent acute cases yield readily to this treatment alone. In the more chronic cases, and where there is a fetid character to the discharge, ten or twelve grains of carbolic acid cryst. may be added to the above with advantage. General treatment by tonics and mercurial alteratives will also have to be resorted to in the more persistent chronic cases before much impression can be made upon them. The following is the mixture which I usually use in these cases:

℞ Tinct. Cinchona. ℥ ii.
Syr. Rhie. ℥ i.
Syr. Glycyrrhiza. ℥ i.
Mix. Hydrarg. Bi-Chlor. gr. i.

A teaspoonful four times a day to an adult.

Or, in many instances, especially where any laryngeal or bronchial complication is apparent, the following mixture will act more efficiently:

℞ Ammonia Hydrochlor., ʒ ii.
Morphæ. Sulph., grs. iii.
Ant. et Potassa Tart. grs. ii.
Mix. Syr. Glycyrrhiza ℥ iv.

A teaspoonful four times a day.

Hydrarg. bi-chlor. one grain can be added, if desired, and would be more especially indicated if there was any syphilitic complication apparent or suspected.

The partial deafness resulting from closure of the eustachian tubes will frequently yield to the use of the inhalation already mentioned. In more severe and chronic cases however, the eustachian tubes may become, more or less, firmly agglutinated together throughout their entire extent. The introduction of the eustachian catheter and the dilation of the tubes by forcing a current of air through them is then necessitated. After dilation in this manner a current of iodized air must be occasionally forced through them by the catheter in order to prevent their becoming again closed.

A very great obstacle and discouragement that is met with in attempting to control these catarrhal affections arises from the fact of their so frequent and persistent recurrence after apparent cure. The membrane lining the nasal passages, remains extremely irritable, and sensitive to atmospheric influences for a long time, especially after being subject to repeated and frequent attacks of catarrh. In a climate like ours, subject at all seasons to the most sudden and extreme variations of temperature and moisture or dryness of the atmosphere, it is almost impossible for those once becoming subject to this affection, to so guard themselves as to prevent the more or less frequent recurrence of fresh attacks. By resorting promptly to treatment each time, however, these attacks can be cut short, and the supervention of any unpleasant sequelæ be prevented.

FUNCTIONAL DISTURBANCE OF THE HEART'S ACTION THE RESULT OF NERVOUS SHOCK.—Mr. R——, aged thirty-five years, a painter by trade, about six months previous to coming under my notice, had fallen from a scaffolding, some fifteen or twenty feet, striking upon his back. No bones were fractured, and no definitely seated injury could be traced beyond the general nervous shock.

He was confined to the bed for some six weeks after the injury on account of severe lameness and stiffness through the spine, especially in the dorsal region. This gradually disappeared however, so that he was able to sit up and get about again. There remained still great general debility, and constantly increasing emaciation, together with a rapid excited action of the heart. The slightest exertion would bring on severe dyspnœa and a nervous, hacking cough.

This condition had persisted up to the time of his presenting himself at my clinic. No curvature or point of tenderness could be detected along the spine. No organic lesion of the heart was apparent on auscultation, neither any evidence of tubercular or other disease of the lungs that would account for the extreme emaciation and hectic symptoms. The functional disturbance of the heart was such as frequently results from a partial failure in the action of the ganglionic nervous system, or some like disturbance of the function of the pneumogastric.

It appeared that in this case, the jar or shock of the fall had so injured the spine as to interrupt or destroy, in great part, the general nerve nutrition. The proper nerve stimulus being thus wanting, muscular atrophy and emaciation naturally followed.

The patient was advised to remain at rest in the recumbent position the greater portion of the time, and to have dry friction applied along the spine two or three times a day. Was to take a light, nutritious diet, as much as the stomach would bear, and as a nutrient tonic, and especially to encourage nerve nutrition, a mixture of Liebig's extract of malt and comp. syrup of the hypophosphite, two parts of the former to one of the latter, was ordered to be taken in tablespoonful doses three times a day.

I was able to keep track of the patient for only about three weeks, but during that time he seemed to be gradually improving.

CONGENITAL MALPOSITION OF THE HEART AND ABSENCE OF THE STERNUM.—A boy nine years of age was brought in by his father for examination and advice on account of general debility and a chronic cough. The child was small for his age, and pale, and emaciated in general appearance. The father stated that he had been sickly from birth, and frequently subject to severe cough and dyspnœa.

Inspection of the chest revealed an entire absence of the sternum. The costal cartilages from either side coming together, formed a cartilaginous septum down as far as the fourth rib. There they divided to form a simple, narrow connecting band between the ribs on either side, and leaving the central space unprotected except by the soft tissues. The chest walls were thin and sunken between the ribs. The apex beat of the heart appeared very distinctly in the centre of the open space opposite the sixth costal cartilage. From that point the heart extended upwards and to the right, bringing the entire organ on the right side of the chest. The space which should have been occupied by the heart on the left side was filled out by the left lung and gave clear resonance on percussion. Further examination revealed, however, extensive condensation and ulceration from tubercular deposits in the upper portion of both lungs. An anodyne and expectorant cough mixture was directed for him, with cod liver oil as a tonic. The chief interest of the case centered in the somewhat rare congenital malposition of the heart.

